# Drug-Resistant Parkinson’s Disease in a Patient With Hereditary Hemochromatosis: A Case Report

**DOI:** 10.7759/cureus.44530

**Published:** 2023-09-01

**Authors:** Conor W Banta, Xavier Zonna, Ronald Lott, Pooja Jaisawal, Amr Elsisi

**Affiliations:** 1 Internal Medicine, Geisinger Commonwealth School of Medicine, Scranton, USA; 2 Internal Medicine, Lake Erie College of Osteopathic Medicine, Erie, USA; 3 Internal Medicine, Guthrie Robert Packer Hospital, Sayre, USA

**Keywords:** drug-resistant parkinson's disease, iron overloaded, preventative medicine, parkinson' s disease, heterozygous hemochromatosis

## Abstract

A 55-year-old male, with a strong family history of hereditary hemochromatosis, presented with progressively worsening right-sided tremor and Parkinsonian symptoms. He was diagnosed with hereditary hemochromatosis based on genetic testing and started undergoing regular phlebotomies to reduce his blood iron levels. Despite extensive trials of different pharmaceutical therapies, including levodopa-carbidopa, his Parkinsonian symptoms were not relieved and continued to worsen. This report serves to highlight the importance of early disease identification and intervention in patients with hereditary hemochromatosis to prevent the development of neurological sequelae, as well as a need for further research into effective therapies in such patients.

## Introduction

Accumulation of iron is a risk factor for cell damage and death. Iron can induce oxidative stress via the Fenton reaction and generate reactive oxygen species that lead to more iron release from cells and further damage potentiating a vicious cycle [1]. In the brain, this oxidation can lead to degeneration of the substantia nigra and ultimately Parkinson's disease (PD) [2]. These findings are often seen in postmortem examinations of brain tissue from PD patients [3]. Alpha-synuclein, commonly produced in PD, is found to disrupt ferritin clearance via autophagy [4]. Hereditary hemochromatosis (HH) is an autosomal recessive disorder of iron metabolism from mutations in the HFE gene (most notably C282Y and H63D) that regulate hepcidin function. This protein serves to control blood iron homeostasis, as well as transport from the intestinal cells that absorb iron. Patients with HH commonly have reduced functionality of hepcidin predisposing them to iron overload in various tissues throughout the body, manifesting through arthritis, cirrhosis, heart failure, or elevated blood glucose. Although not as common, the association between brain iron accumulation and neurodegenerative disease has also been proposed in recent literature [1-3]. Patients with HH may be at an increased risk of PD due to their dysregulation of iron storage and metabolism. We present a case of a patient with symptomatic HH and concurrent treatment-resistant PD to promote early screening by clinicians, as well as raise awareness for the increased need for alternative PD treatments for these patients.

## Case presentation

A 55-year-old male, with a past medical history of dyslipidemia, hypertension, gout, and a strong family history of HH (one symptomatic first-degree relative, as well as carrier first and second-degree relatives), presented to the outpatient clinic with a one-year history of progressively worsening right arm tremor. Physical exam at initial presentation revealed a fine right arm tremor at rest with no rigidity, muscle atrophy, or gait changes. A two-month trial of propranolol at various doses, as well as primidone, failed to improve the tremor, which led to a presumptive diagnosis of early-stage PD. Consequently, a trial of pramipexole was initiated. During this time, the patient underwent genetic testing, which revealed that he was a compound heterozygote for C282Y and H63D genes for HH. Pramipexole failed to improve the right arm tremors, and the patient continued to experience worsening tremors, malaise, and fatigue. Regular therapeutic phlebotomy was initiated, with peak ferritin levels of 1,014.0 ng/ml recorded. As the patient was eligible for a genetics consultation, a referral was completed. However, the patient opted not to pursue further investigation and chose to undergo regular ferritin monitoring. During the following months, the patient also developed several cardinal signs of HH, including new-onset skin hyperpigmentation, type 2 diabetes mellitus, and arthralgia.

**Figure 1:**
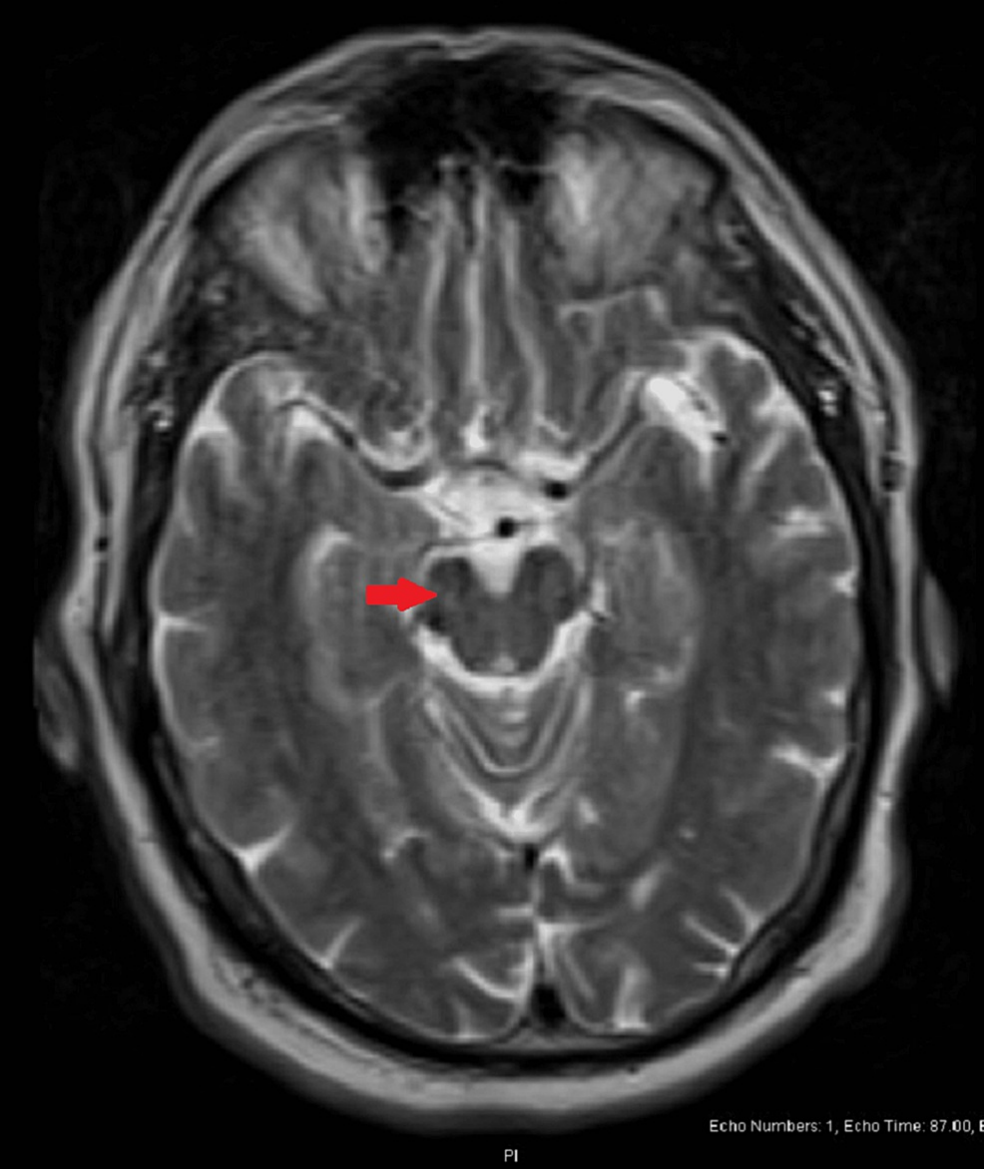
Axial T2-weighted MRI shows right-sided substantia nigra hyperintensity loss (arrow) Image subject to motion degradation MRI = magnetic resonance imaging

Despite treatment with pramipexole and periodic therapeutic phlebotomy, the patient's tremors continued to worsen. The patient was also trialed on ropinirole without improvement. At additional follow-up, the patient reported micrographia and difficulty eating with his right hand, with new head tremors, right arm cogwheel rigidity, and decreased right arm swing while walking. A brain MRI revealed a loss of intensity in the substantia nigra on the right with indeterminate findings on the left, although no high-resolution 3-D SWI imaging or dopamine transporter scans were performed (Figure 1). Additional testing demonstrated normal ceruloplasmin, TSH, and T4. 

As the patient's Parkinsonian symptoms continued to worsen, a trial of fast-acting propranolol and low-dose levodopa-carbidopa was started with no improvement noted. Over the course of several months, the patient continued to develop symptoms such as new-onset pill-rolling tremor, right-sided bradykinesia, decreased blink rate, neck tremors, and slow, shuffling gait that significantly impacted his activities of daily living. As a final effort to treat his Parkinsonian symptoms, he was switched to high-dose levodopa-carbidopa therapy and referred to a movement disorder specialist at a tertiary care center, who agreed with the diagnosis of PD and recommended a trial of amantadine.

## Discussion

HFE homozygosity for the C282Y mutation comprises the majority of cases of HH [5], while HH due to the C282Y/H63D heterozygosity is relatively uncommon [6]. This case demonstrates a rare compound heterozygosity found to be present in approximately 2% of the US population [7]. A 2022 retrospective analysis of a Canadian population found that only 4% of these compound heterozygote patients had iron overload at baseline, and this only increased to 5.3% at 10 years, concluding that this was a low penetrance genotype [6]. The rarity of this presentation is further shown in a study of European genetic populations, which concluded that iron-related disease is very rare for this heterozygosity [8]. Therefore, this patient becoming symptomatic with diabetes and arthralgia with an elevated ferritin level is unusual for this genotype. 

Although the research between HH and PD is limited, a link may be present and warrants further investigation. A study of four separate patients with concurrent HH and symptomatic PD concluded that iron overload may disrupt the blood-brain barrier and facilitate iron deposition in the CNS [9]. This information has been reproduced in larger-scale studies as well. A 2003 study found that patients with PD were significantly more likely to be homozygous for C282Y and that being a carrier also increased the risk for non-PD Parkinsonism compared to controls [10]. A study of a Portuguese cohort also found an increased frequency of C282Y carriers in the PD group as compared to controls [11]. This may have implications for clinicians as early recognition and treatment of iron overload may prevent neurodegeneration. Clinicians may also perform more frequent focused histories and neurological screening examinations in HH patients to monitor for neurodegeneration.

Neurological symptomatology of PD is complex. Typical symptoms include tremors, movement difficulties, gait disturbances, and muscle rigidity. Atypical syndromes can involve psychiatric disturbances, as well as difficulties with gaze, autonomic function, and sensation. A 2018 systematic review of 50 papers found seven papers describing both typical and atypical Parkinsonian symptoms associated with HH [12]. These studies were also all associated with Parkinsonian symptoms that were unresponsive to treatment with traditional pharmacologic therapy such as levodopa [12]. This pattern was corroborated in our patient, where dopamine agonists and levodopa-carbidopa did not alleviate his neurological symptoms. However, this patient largely showed typical PD symptoms, such as tremors, bradykinesia, and shuffling gait. This information further advocates for increased research into finding effective treatments for patients with neurodegenerative disease as a sequela of iron overload. Researchers are currently suggesting further studies on iron chelators in the treatment of diseases such as PD and AD due to the association of heavy metal deposition and neurodegeneration [13]. A 2014 randomized double-blind study found that deferiprone improved treatment outcomes and delayed disease progression in PD patients while also decreasing iron deposition in the substantia nigra [14]. Although further research is needed, these initial positive results may be indicative of the potential future direction of HH treatment for the prevention of neurodegenerative diseases.

## Conclusions

Iron accumulation and associated neuronal toxicity places patients with HFE mutations at an increased risk for neurodegenerative diseases such as PD. This risk may be present in patients of varying HFE genotypes, including C282Y homozygotes, H63D homozygotes, or compound heterozygotes. Increased neurodegenerative disease risk in these patients coupled with a lack of responsiveness to traditional PD therapies highlights the need for future research directed toward the prevention and management of hereditary hemochromatosis-related neurological disorders.
